# A global framework for a systemic view of brain modeling

**DOI:** 10.1186/s40708-021-00126-4

**Published:** 2021-02-16

**Authors:** Frederic Alexandre

**Affiliations:** 1 INRIA Bordeaux Sud-Ouest, Talence, France; 2 Institute of Neurodegenerative Diseases, University of Bordeaux, CNRS UMR 5293, 146 rue Leo Saignat, 33076 Bordeaux, France; 3 LaBRI, University of Bordeaux, Bordeaux INP, CNRS UMR 5800, Talence, France

**Keywords:** Brain modeling, Cognitive functions, Memory system

## Abstract

The brain is a complex system, due to the heterogeneity of its structure, the diversity of the functions in which it participates and to its reciprocal relationships with the body and the environment. A systemic description of the brain is presented here, as a contribution to developing a brain theory and as a general framework where specific models in computational neuroscience can be integrated and associated with global information flows and cognitive functions. In an enactive view, this framework integrates the fundamental organization of the brain in sensorimotor loops with the internal and the external worlds, answering four fundamental questions (what, why, where and how). Our survival-oriented definition of behavior gives a prominent role to pavlovian and instrumental conditioning, augmented during phylogeny by the specific contribution of other kinds of learning, related to semantic memory in the posterior cortex, episodic memory in the hippocampus and working memory in the frontal cortex. This framework highlights that responses can be prepared in different ways, from pavlovian reflexes and habitual behavior to deliberations for goal-directed planning and reasoning, and explains that these different kinds of responses coexist, collaborate and compete for the control of behavior. It also lays emphasis on the fact that cognition can be described as a dynamical system of interacting memories, some acting to provide information to others, to replace them when they are not efficient enough, or to help for their improvement. Describing the brain as an architecture of learning systems has also strong implications in Machine Learning. Our biologically informed view of pavlovian and instrumental conditioning can be very precious to revisit classical Reinforcement Learning and provide a basis to ensure really autonomous learning.

## Introduction

As stated by the French biologist and physician Henri Laborit [60], the motivation of living beings is being, i.e. maintaining their organic structure. Whereas this statement is obvious for basal animals, it is too often neglected when studying high-level cognitive functions, particularly in humans. Most of the time in computational neuroscience, such cognitive functions are associated to specific regions of the cortex and not to the bodily dimension or even to subcortical structures. Their characteristics are described as resulting from purely cortical dynamics, with no references to motivational or emotional groundings. In neuroscience, some authors and paradigmatic approaches have stressed the limitations of such corticocentric views, compared with a myopia [89], minimizing the essential role of subcortical structures. On the modeling side, the domain of embodied Artificial Intelligence has shown through robotic experiments [98] that complex behaviors may result from elementary loops between sensors and actuators, exploiting the properties of the body instead of a complex representation of information (throughout this paper, we use a classical definition of information carried by data [spikes in the neuronal case], as a contextual interpretation of the data, providing a meaning, as it will become more clear when fundamental questions will be introduced below. It is for example proposed in [113] that the non-homogeneous distribution of sensors in the retina can explain some visual target selection principles in a more parsimonious way than purely cortical mechanisms.

More fundamentally and anchored in cognitive science, enactivism, the theory of enaction [118], stresses principles like autonomy and ecological meaning of the behavior. In this theory, autonomous behavior is a central characteristic and is considered at different time scales. Fundamentally, a living being must choose on its own and at each moment the most adapted behavior and can only rely on previous learning (ontogeny) and on pre-established abilities (phylogeny, seen as learning at a long time scale). Ecological meaning refers to the motivational and emotional bases of behavior that have to be taken into account. Cats chase mice, because they have such motivations, needs and goals—because they are cats.

In spite of their important role to define needs of the body and goals to be reached, the motivational and emotional dimensions of behavior are little studied in computational neuroscience and in cognitive science. Building not only on interoceptive information like visceral signals but also on somatosensory information (pain, pleasure, temperature), the insular cortex is hardly considered in cognitive modeling studies though reported to play a central role in defining motivations of the body to act, like feeding, breeding, preserving the integrity of the body [21]. Biologically significant events important for survival signaled not only by such interoceptive signals but also by sensory information (e.g. related to the perception of a predator or of social signals) can be associated by learning with other neutral events that will elicit emotions useful to anticipate the former ones and to detect goals to be pursued or avoided. Gros [43] suggests a specific role of information of reduced complexity for emotions that can become conscious feelings, also described as mental experiences of body states [23].

Such a body of principles should make humans more conscious of their animal condition. It underlines the strong links between the brain, the body and the environment and, within the brain, is a strong motivation to consider large brain loops instead of cortical regions in isolation and to consider the multiple learning mechanisms at work within these loops. At the functional level, this is also a plea for defining a global cognitive architecture in which any cognitive operation in consideration should be delineated. Decision making, planning, selective attention or perceptual identification should not be studied, and models of the corresponding cerebral circuitry should not be elaborated, without a reference to a global framework relating cognition and the brain, seen as a whole and in relation to the body and the environment. Else, the risk is to just study mechanisms apart from the rationale for their existence and consequently to forget some of their fundamental characteristics and resources.

In agreement with these considerations, we present here in a systemic view, a general framework of brain organization that has been elaborated from the analysis of the literature in cognitive, experimental and computational neuroscience. It is intended, for future-specific studies of brain-inspired cognitive mechanisms, to serve as an outline in which each of these studies should be placed, for a better understanding of its contribution in general cognition and for consistency in this systemic view of cognition that we affirm here to be essential.

**Fig. 1:**
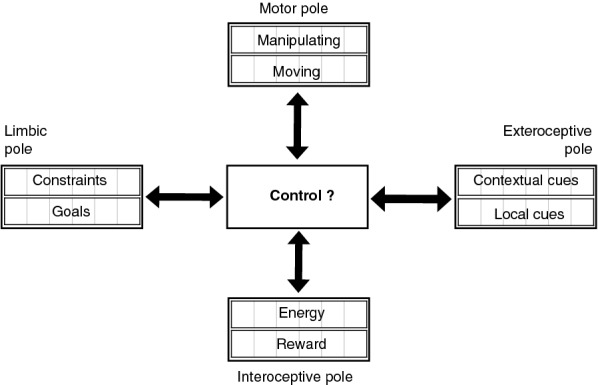
Information flows to control an intelligent agent. Controlling the behavior of an intelligent agent can be organized under several poles of information. In a perceptual (or exteroceptive) pole, the agent can perceive local cues from its environment (shape, color of objects) and more global contextual cues (position and organization of objects in the environment). In a motor pole, the agent can trigger actions to move or to manipulate objects. To demonstrate an intelligent behavior, some goals (objects to reach) and constraints (situations to avoid) must be specified, as it is proposed in a limbic pole (the use of this term is explained later in the paper). Taking into account goals and constraints can be monitored and possibly learned if some information like rewards and levels of energy are given, gathered here in an interoceptive pole

Based on strong neuroscientific and cognitive bases, this framework might be useful to help scientists in these domains have a more general view of the “big picture” necessary to develop a brain theory as well as to give a global context to cognitive modeling. It is particularly destined for modelers in computational cognitive neuroscience, with not necessarily a strong background in neuroscience, that would like to have a global and functional view of the cognitive architecture and its corresponding cerebral circuitry, within which they might display the topic on which they are presently working on. Generally, such scientists begin with a global view of a task, where the problem is to control an intelligent agent in its environment. As sketched in Fig. 1, this problem can be specified with several information flows organized under different poles: the perceptual and motor characteristics of the agent are, respectively, described in input and output information flows called the exteroceptive and motor poles, whereas task instructions are given to the agent in a limbic pole gathering specified goals and constraints and can be monitored within an interoceptive pole, through internal sensations corresponding to rewards, punishments and levels of energy. The aim of this paper is to propose a structured and comprehensive framework to help implement such a task or a part of it with a clear understanding of how it is inserted in a global cognitive architecture.

In the following, we present and motivate on a neuroscientific basis the main ingredients of this framework and we explain how it can be used to implement the task. In Sect. 2, we specify the mentioned information flows and their links to the bodily dimension of cognition and to its emotional and motivational anchoring. This leads to two important characteristics of the framework: the central role of Pavlovian and instrumental conditioning in the organization of behavior and the structuring role of four fundamental questions for defining the control of behavior. In Sect. 3, we explain how these four questions are addressed in sensorimotor loops along increasingly complex organizational principles and under a phylogenetic perspective. In Sect. 4, we propose that, along evolution, three complementary learning mechanisms are added to the two kinds of conditioning, to create a system of interacting memories, able to cope with more complex situations and we introduce accordingly cerebral structures and circuits implementing this dynamical system. We conclude this paper by discussing implications of this framework both in brain and modeling sciences.

## Main information flows

As mentioned above, even high-level cognitive functions should be studied by taking into account their bodily, emotional and motivational dimensions. We make this point clear here by describing more precisely the information flows and poles of information mentioned in Fig. 1. In Sect. 2.1, we explain that they are the basis of the relations between three worlds to consider in cognitive functions (brain, body, environment). We provide in Sect. 2.2 a bodily grounding to emotional and motivational learning and introduce the corresponding adaptive schemes and their central role in behavior. Importantly here, this informational structure is also a natural way to introduce in Sect. 2.3 four questions to consider to implement a task.

### Three worlds to conciliate

The brain is facing complex and dynamic worlds, on each of which it can sense information and it can act, possibly resulting on modifications in that and other worlds. Of course, one of these worlds is the brain itself, often represented as a set of numerous processing units, each collecting information from some units and generating activities to modify others. In addition, it is often underestimated that the brain communicates with three other worlds. We call these worlds the external environment, the extended body and the internal body. The external environment corresponds to the external world, including objects subject to the laws of physics and beings also subject to the laws of nature, possibly including intentionality. These objects and agents exist in space and time and can be sensed by external sensors (i.e. seen, heard, touched, tasted or smelt), defining perception. The extended body considers the body as an agent in the external environment, in which it may act. The extended body is composed of parts (e.g. limbs, head) carrying the external sensors. Their positions in space can be sensed by proprioception and can be modified by elementary actions and integrated motor programs (e.g. walking, grasping, speaking). We call exteroception these external flows of information (perception and proprioception). The internal body refers to all the machinery that makes the body work internally at the visceral, chemical, hormonal levels, i.e. eat, drink, breath, digest, etc. This defines the fundamental needs of a being, depending on internal states that can be sensed by interoception. Homeostatic mechanisms and other internal and external responses can modify these states.

**Fig. 2:**
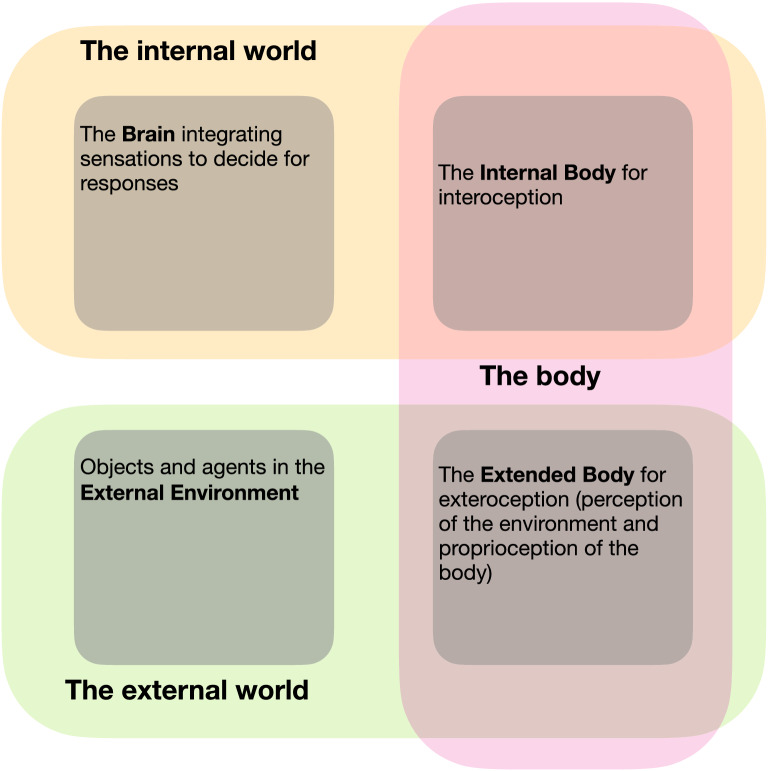
Three worlds to conciliate. In an enactive view of cognition, the brain is not seen in isolation but in systemic inter-relation with the external world and with internal states. This leads to the specification of two facets of the body. The extended body interacts with the external environment through exteroception (proprioception and perception) and through external responses. The internal body feels internal states by interoception and can trigger internal responses. Altogether, this defines the sensory and motor information flows of a brain + body system, acting and learning in the external environment to satisfy some needs, expressed as internal states

As sketched in Fig. 2, the brain has consequently exteroceptive (perceptual and proprioceptive) and interoceptive sensors to get information about these worlds and their inner dynamics. It can act on them through a series of processes that we will call responses at large. These responses can be voluntary or involuntary and be applied to the extended or internal body, respectively, corresponding to motions of parts of the body with skeletal muscles (e.g. speaking or running) and to the activation of endocrine or exocrine glands (e.g. releasing an hormone or crying), of smooth muscles and of the heart. Impacts of these responses in the three worlds can also be perceived by sensors.

For both motor and sensory aspects, the central nervous system (including the brain) is connected to these worlds by the peripheral nervous system, including a somatic part (for perception and proprioception and for external responses) and a visceral part (also known as autonomic nervous system, for interoception and internal responses). The autonomic nervous system is itself divided in two parts, the parasympathetic system responsible for the feed and breed activities and the sympathetic system for the fight or flight activities. In a simplistic view, this can be categorized in a dichotomic way with positive (pleasant) situations and incentives to exploit them for the nourishment and the reproduction of the body, opposed to negative (unpleasant or painful) situations and incentives to get rid of them for the integrity of the body. As we will discuss later, though partly overlapping, it would be too simple to directly associate this dichotomy to rewards and punishments, since for example a lower punishment than expected can be seen as pleasant. This can be more nicely integrated in a two opponent process system [29] with mutual inhibition between two classes with opposite properties, acting against a baseline.

Before describing more technically information processes in the brain, it is fundamental to stress again that they have been selected in an evolutionary scheme, particularly to enable living beings to maintain their structure, to optimize survival and reproduction. This sets a special emphasis on the internal body world that has been designed and complexified by evolution to represent special body states indicating critical situations (that we will call emotions below) and giving specific incentives for that aim (that we will can motivations). We will define accordingly in Sect. 2.2 below, the two behavioral processes in charge of selecting responses, based on these body states, namely pavlovian and instrumental conditioning. In both processes, signals that are received can be used to directly trigger responses, based on their intrinsic value or on their capacity to activate internal representations. They can also be used to modify internal representations or to create new ones, following several learning processes that will be described in the next sections.

In summary, the processes for the transduction of signals into responses and for the elaboration of internal representations of information are based on the signals received from the three worlds, on the current state of the memories and on the architecture of the cerebral structures.

### Pavlovian and instrumental conditioning

Considering the brain as a system integrating different kinds of sensations to decide for different kinds of responses, as sketched in Fig. 2, several mechanisms of increasing complexity have been aggregated to this system along evolution. A first set of mechanisms directly associated to emotional learning is related to pavlovian (or respondent) conditioning [7]. Some biologically significant stimuli also called Unconditional Stimuli US (e.g. a predator or some food) can be automatically identified (without learning) and trigger Unconditional Responses UR (e.g. freezing or salivation). Pavlovian conditioning corresponds to learn that some initially neutral Conditional Stimuli CS (e.g. a tone or a light) announce the arrival of US. Pavlovian conditioning has been modeled by learning rules modifying CS–US associations as a function of prediction errors between the actual US and the US predicted by the CS [61]. If several CS predict a US, the more reliable predictors are taken into account (automatic processing, Mackintosh rule). In case of a US not explained by the CS, learning rather applies on new predictors (controlled processing, Pearce-Hall rule). Subsequently, the occurrence of CS can generate two phases of behavior [7]. In the consummatory phase, the CS is associated to the sensory properties of the US and triggers specific responses like chewing or blinking. In the preparatory phase, if the CS is distant, it is associated to motivational properties of the US and to its valence (aversive or appetitive) and triggers non-specific responses such as arousal, heart rate increase and approach.

At this stage, it can be interpreted that pavlovian learning is a way to anticipate, upon CS arrival, the negative or positive characteristics of the US and to prepare the body to this inevitable event. In the pavlovian scheme, responses are stereotyped (also called pavlovian reflexes) and are consequences of the learned associations. Several mechanisms have been described, inserting other responses in the pavlovian process [7]. In autoshaping, an action can be triggered to more easily get a CS. In pavlovian instrumental transfer (PIT), animals exposed to a CS associated to a US trigger more frequently the (instrumental, cf. below) response that was learned to obtain that US. Similar to the distinction between specific and general responses evoked above for consummatory and preparatory phases of behavior, PIT can be built on the specific sensory features or on the general affective properties of the US [7]. We will explain in Sect. 4.3 that these two kinds of processing are in fact mediated by different circuits, serving different purposes.

Whereas, based on CS, pavlovian conditioning passively predicts the US to occur and prepares the body to this event, another more active learning scheme called instrumental (or operant) conditioning considers that, by acting, some reinforcement might be obtained. In this case, the learned association is between a response and the outcome observed to be the consequence of the response. This principle is also defined as Thorndike’s law of effect: A response will be triggered more (resp. less) frequently when observed to lead to a positive (resp. negative) consequence. Alternatively, a response can be triggered more frequently if it leads to the avoidance or removal of a negative stimulus. The two opponent process system mentioned above is a good basis to consider these negative cases where, at the end, no reinforcement is given: the absence of negative outcome can be considered as a positive outcome, on which to build learning with so-called conditioned inhibitors. It has also been associated to the representation of safety [40].

Since here, the response is voluntary, it is possible to consider the corresponding level of need, devaluate the outcome and refrain from acting if the motivation is low. Whereas pavlovian conditioning simply defines how much an outcome is liked, instrumental conditioning considers how much it is currently wanted and chooses to trigger responses taking motivations into account. This can be extrinsic motivations, to get a desired (external or extrinsic) outcome satisfying fundamental needs, including integrity of the body, seen as a positive motivation in the framework of the two opponent process system. With the elaboration of more complex internal representations that will be described below, expressing intrinsic motivations [85] will be also observed. They are related to a more abstract need of (intrinsic) information, to obtain from the exploration of the complex world and from the monitoring of internal activity, as it is the case with curiosity and attention toward novelty.

Instrumental conditioning can be performed under the control of (or conditional to) stimuli also called occasion setters, that can become conditioned reinforcers [17], leading to chaining in complex behavioral goal-directed sequences toward primary reinforcers (respectively, defined as subgoals and goals in planning). Conversely, these associations can be transformed in habits through extensive learning, where the conditional stimuli directly elicit responses without references to the outcomes to be obtained [12]. More generally, this refers to a dichotomy between goal-driven behavior (where the behavior is driven by internal goals and can adopt complex schemes) and stimulus-driven behavior (where the agent mainly reacts to perceived stimuli).

### Four questions to be addressed

We have seen above that it is important to identify stimuli (or ‘objects’) in the world as possible goals of behavior (emotional or Pavlovian learning) and relate them to the corresponding need they can satisfy, to decide if it is worth triggering responses to get them (motivational or instrumental learning). But in case of more complex scenarios, it is also important to learn how to reach these objects and how to act on them, to implement preparatory and deliberative phases before decision. We will explain in Sect. 3.1, how these elementary behaviors are implemented in subcortical structures and in Sect. 3.2, how we arbitrate between them, also with the help, as described in Sect. 4, of additional learning capabilities in other cerebral structures. We propose a functional introduction to these elementary behaviors and sketch them as four fundamental questions: what, why, where and how. Here, for illustration, we relate each of these questions to a cortical area associated to the underlying information representation.

The why and what questions relate the internal body and external environment. The what question is a way to encode a CS and its emotional impact. The CS can be seen as the current goal of the behavior (for example a bottle of water). It is consequently important to encode its physical characteristics (shape, color) to be associated to its emotional value (preference, like, dislike). Several regions of the posterior ventral (or temporal) cortex have been reported to be selectively responsive to such physical characteristics [102]. The why question corresponds to the characteristics of the motivational impact on the body and is useful to encode the impact of the US or the bodily cost of a response to get it. Consider for example the level of water deprivation or the intensity of a pain. Such information is represented in the posterior insular cortex [21]. It can motivate the behavior, explaining ‘why’ we act (for which purpose) and why (up to which level) we accept to spend our energy.

The where and how questions relate the extended body and the external environment. Answering the where question provides information about the position of an ‘object’ and particularly with regard to (some parts of) the body. It will be important to locate and orient toward it. The how question refers to the need to learn how objects can be modified (e.g. approached, moved, manipulated) by the action of some body parts. The posterior dorsal (or parietal) cortex has been reported to be involved in both where and how functions [72], respectively, in its inferior and superior parts.

These cortical areas were just mentioned for illustration, because as it will be detailed in Sect. 3.1 below, many other brain regions are involved in answering these questions. Structuring under these four questions provides the main ingredients of a simple goal-directed behavior: we evaluate why it is important to satisfy a need; accordingly, we describe the goal of our response (what are its characteristics) and locate it (where) for consumption (how). But of course, in the real world, things are not so easy. Several motivations and goals can be in competition. Their characteristics can be difficult to extract. Variable delays can exist between the main ingredients of the behavior (the US, CS and responses) and some abstract reasoning can be needed (the bottle of water is in the fridge and I have to find the kitchen beforehand). All these elements correspond to increasingly complex behaviors, made possible along evolution, as we describe in the next section.

## Increasingly complex behaviors

**Fig. 3:**
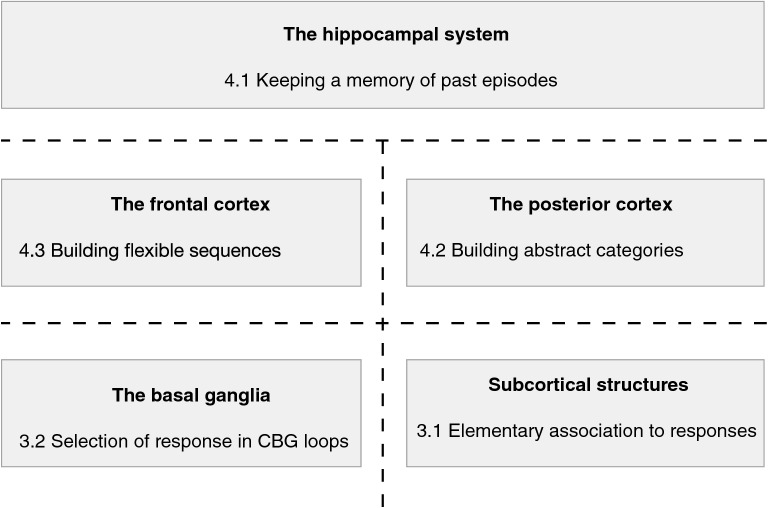
Organization of the paper around five cerebral structures. For the description of functional and learning mechanisms in the brain, the remaining of the paper is organized in five sections, each centred on a cerebral structure and a computational mechanism

It is not intended here to reactivate the outdated triune brain theory [68], promoting a purely hierarchical description of brain structures and functions through three stages of evolution each bringing more evolved behaviors (in short: reflexes, emotions and motivations; abstract thinking). Instead, it will be clearly explained here that both on the sensorimotor [107] and emotional and motivational [17] sides, complex and bidirectional relations exist between recent and older cerebral structures, organizing a variety of control loops with different constants of time. For the sake of clarity, it is proposed to refer to a series of evolutionary steps to organize the remaining of the paper as described in Fig. 3, keeping clearly in mind that these steps are very schematic and that, of course, in addition to the initial function of some structures evoked here, evolution has continued in all parts of the brain including in older structures, resulting in the imbricated control loops mentioned just above and decomposed in more details below.

### Elementary association to responses (the role of subcortical structures)

At an elementary level, key ancient subcortical structures, already existing in basal animals without a cortex, can be linked to the four questions mentioned above, directly associating responses to rough representations of sensory information.

*What—The amygdala* The amygdala is a heterogeneous set of structures with sensory and motor aspects [112]. Among its nuclei [62], the lateral nucleus receives a wide spectrum of sensory inputs from the thalamus and the cortex and is generally reported as a place for storing CS–US associations. The central nucleus of the amygdala (CeA) is the main output region for the expression of innate emotional responses and related physiological responses, particularly in relation to the periaqueductal gray (PAG) and the lateral hypothalamus for resp. aversive and appetitive behaviors. Another major nucleus is the basal nucleus, particularly in charge of information exchange with higher level structures like the prefrontal cortex and the hippocampus [18]. Neurons in this nucleus encode a variety of information for aversive and appetitive stimuli, related to the sensory nature of the US, to conditioned inhibitors and, for instrumental conditioning, related to conditioned reinforcers [10]. It also encodes the level of arousal, ambiguity and unpredictability of information [94]. Altogether, the lateral and basal nuclei, also called the basolateral complex (BLA), is both a place for representing the valence and the value of emotional stimuli and a relay for more elaborated processing in the cortex, ventral striatum and hippocampus, in direct association with the central nucleus CeA responsible for simple pavlovian responses and also involved in more elaborated emotional responses [17].

*Why—The lateral hypothalamus* The lateral hypothalamus contains nuclei evaluating needs of the organism and responsive to appetitive US, and nuclei promoting responses related to digestive functions, blood pressure and other visceral functions [22]. In the framework of the two opponent process system, it is also strongly linked to the PAG for pain control. Similarly, on the aversive side, the PAG also encodes corresponding US and mediates defensive responses [9]. Both structures are consequently reported as low-level homeostatic centres and are involved in preparation of the sensory inputs to the insula and in the expression of more elaborated motivational behaviors [17]. Similar to other subcortical structures depicted here, these structures, initially organized in sensory and motor domains to promote direct associations from visceral and somatic signals towards simple appetitive and aversive behaviors, have evolved in association with more recent structures to enrich their representations and participate within more complex circuits to more elaborated behaviors.

*Where—The superior colliculus* The superior colliculus (also called the tectum in basal animals) is a structure mainly studied for its involvement in eyes movement and gaze orientation [63]. It is composed of several layers, some receiving mainly visual information from many regions in the brain, including directly from the retina. The more superficial sensory layers are topographic maps of the surrounding environment and are in direct association with deeper motor layers for eye movements towards the place elected by competition in the sensory layer [113]. It has been remarked that this structure can also perform direct sensorimotor associations for orientation of the whole body for tracking novel stimuli, for defensive movements and flight in case of a danger [26]. For more complex oculomotor behavior, the superior colliculus remains an essential stage between the retina and the posterior dorsal cortex and the Frontal Eye Field FEF [107] in the frontal cortex.

*How—The cerebellum* The cerebellum is a cerebral structure known for its role in sensorimotor control [66]. Its granular cells are sensory inputs arranged in somatotopy and receiving most kinds of sensory information including from proprioception. They are directly associated with Purkinje cells projecting to all cerebellar output nuclei targeting motor systems responsible for motor control, from movement execution to planning. Particularly, these circuits have been shown to be involved in limb movements, manipulation, speech, both for direct automatic movements (postural adjustment, balistic movements, conditioned reflexes) and for the control of voluntary movement and even more abstract cognitive functions, through higher level centres [70].

**Fig. 4:**
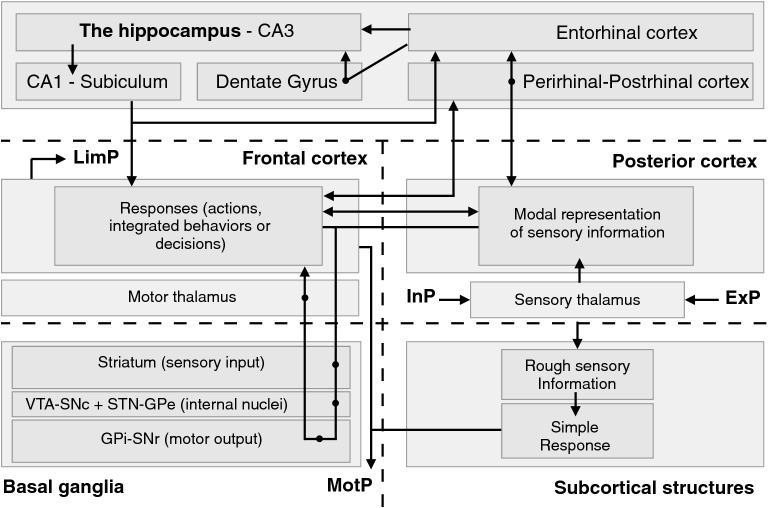
Generic circuitry within and between the five cerebral structures. Organized around the five cerebral structures introduced in Fig. 3, this figure introduces their sensory and motor aspects and the main circuitries described in the corresponding sections in the paper. Black dots on arrows represent intermediate steps in a circuit. The main inputs and outputs of the control problem considered here (cf. Fig. 1) are also introduced (ExP: exteroceptive pole; InP: interoceptive pole; LimP: limbic pole; MotP: motor pole). Importantly and as a major element in this paper, these circuits are generic and will be similar for each region of these structures addressing a specific question, as it will be reported in Fig. 5. Note also that in the present figure and for the sake of clarity, relations between subcortical structures and the striatum as well as the hippocampus are not represented. See text for details

In summary and in accordance with an enactive view of cognition, it is important to consider why the behavior is triggered, toward what goal, where it is situated and how it can be accessed. Each of these questions can be tackled independently by a simple sensorimotor association and we have reported here evidences that, for each question, one cerebral structure is particularly involved in elaborating such simple association. We have also indicated that, in each case, other higher level structures can build more complex relations on the association, in a classical framework of imbricated sensorimotor loops [45], convenient both for incremental learning and for responding at anytime, as sketched in Fig. 4. Before bringing more information about other levels of imbrication, we first evoke another problem, related to a need of consistency between the selection of answers to these questions.

### Selection of response in Cortex-Basal Ganglia (CBG) loops

In superior mammals, the control of action is mainly cortical. In summary and as represented in Fig. 4 (cf. Sections 4.2 and 4.3 for details), the cortex can be separated in regions encoding sensory information (that we will call posterior cortex for simplicity, because most of them are posterior to the central sulcus) and in regions encoding information related to responses (elementary actions, integrated behaviors or decisions impacting {acting on} internal variables) in the frontal cortex [39], anterior to the central sulcus. Section 4.3 explains how these responses in each region of the frontal cortex are learned, executed and monitored as transitions between sensory states in associated regions of the sensory cortex. Some frontal regions are agranular (with no or minor layer IV). They correspond to the motor and premotor cortex and to a part of the orbitofrontal cortex. They perform a selection of response from perceptual cues and internal state values when external contingencies are stable. This corresponds to stimulus-driven and habitual behavior [12]. Other frontal regions, called together the prefrontal cortex (PFC), are granular and are only present in primates (though this is disputed [116], as some features of the prefrontal cortex might be present ini rodents). They correspond to the ventral and dorsal regions of the medial prefrontal cortex (mPFC) and of the lateral prefrontal cortex (lPFC) and to the frontopolar cortex FPC, unique in humans [56]. The prefrontal cortex is engaged in executive (or cognitive) control with such mechanisms as Task Sets and Working Memory described in Sect. 4.3. In summary, when the world is uncertain or when the behavior is guided by internal goals, the idea is to replace the dominant default behavior guided by stimuli, by the selection or the design of new rules (addressing specific tasks), from the retrospective or prospective evaluation of the situation.

In both cases (response or rule selection), the goal is to make a decision from limited resources in the motor case (we cannot at the same time consume pieces of food located in two distinct places) and in the cognitive case (the distributed cortical circuitry prevents from easily mixing pieces of information that should be aggregated for a complex decision). P. Redgrave and colleagues have nicely addressed this problem in [100] and have proposed that the basal ganglia (BG) is the cerebral structure responsible for addressing this problem of limited resources, underlining that, even if brain processing is generally distributed, the process of response selection is fundamentally centralised, which is rather rare in brain functioning.

The inner processing of the BG is very complex, involving a variety of internal structures, pathways and mechanisms, as evoked in [99], which are still topics of intense research. Basically, the BG can also be described as a sensorimotor set of nuclei, with, on the sensory side, the striatum as an input structure receiving sensory and motor information and, on the motor side, the internal part of the globus pallidus and the substancia nigra pars reticulata (called together GPi-SNr in primates; can also correspond to other names in other species) acting as output motor structures. Most of the models of response selection in the BG [12, 20, 48] describe inhibitory and excitatory pathways associating internal BG nuclei (like the external part of the globus pallidus, GPe) and defining loops between the BG and the Cortex, called CBG loops and sketched in Fig. 4.

For each CBG loop, a region of the striatum receives afferent information from frontal and posterior cortical regions and one among the responses represented in the frontal region is going to be selected by a funneling effect from the striatum to GPI-SNr and back to the frontal cortex after a thalamic step [2]. Competition between the internal inhibitory and excitatory pathways is responsible for either maintaining the current selection (maintaining a working memory or the execution of a response) or changing and updating the selection. This process of response selection is generally permitted by a kind of reinforcement learning, with a prominent role for the dopamine, sent by the ventral tegmental area (VTA) and the substantia nigra pars compacta (SNc) to modulate cortico-striatal connections. This process helps define elementary associations (the subtle combinations of cues, responses and contexts permitted by this circuitry that we call here rules) that minimize reward prediction errors [53]. In addition to this fundamental role of dopamine in learning, another synergistic role of dopamine related to the control of performance also participates in the gating mechanism, deciding for the maintenance or updating of information and will be described in Sect. 4.3.

**Fig. 5:**
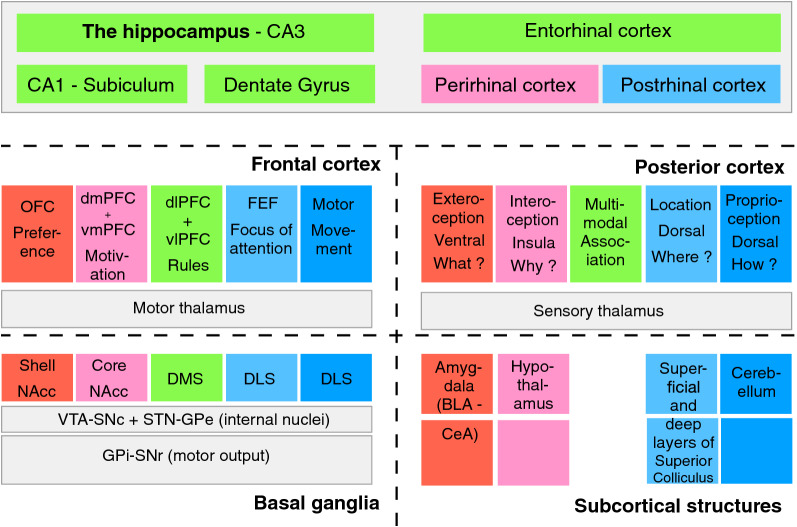
Five loops associating the five cerebral structures. This scheme of the brain underlines some important anatomical and functional characteristics to better understand how information flows are processed in the brain. It is proposed that five kinds of neuronal structures bring more and more complexity and flexibility along phylogeny: (i) subcortical structures (the Amygdala (and its inner nuclei, the basolateral complex BLA and the central nucleus CeA), the Hypothalamus, the Superior Colliculus (with its superficial and deep layers) and the Cerebellum), (ii) the Basal Ganglia (with the striatum composed of its dorsolateral part DLS, dorsomedial part DMS and ventral part, also called Nucleus Accumbens NAcc with a shell and a core division; with output structures, the internal Globus Pallidus and the substantia nigra pars reticulata GPI-SNr and internal nuclei, STN (subthalamic nuclei) and GPe (external Globus Pallidus); with dopaminergic regions, ventral tegmental area VTA and substantia nigra pars compacta SNc); (iii) the hippocampus with its main internal structures dentate gyrus DG, CA3 and CA1 and its associated cortical structures (the entorhinal, postrhinal and perirhinal cortex); (iv) five regions of the sensory cortex receiving inputs from the sensory thalamus (the ventral, dorsal, associative and insular cortex); (v) five regions of the frontal cortex including the orbitofrontal cortex OFC, the ventral and dorsal parts of the medial prefrontal cortex vmPFC and dmPFC, the lateral prefrontal cortex with ventral and dorsal parts vlPFC and dlPFC, the frontal eye field FEF and the motor and premotor regions. As explained in more details in the text, colors refer to the major implication of certain regions in these structures, to answer fundamental questions for the selection of goals (what goal and why, respectively, in red and pink) and for their spatial access (where and how, respectively, in light and dark blue), whereas the green color is for associative processes. These colors also refer to preferential projections between these regions, particularly forming five CBG loops between the structures, even if the text also explains that some complex functions result from interactions between different loops. The circuitry within each loop is similar and depicted in Fig. 4

Several parallel loops (five in [2]) have been described between the cortex and the BG, adapting this generic function of response selection to different kinds of information. But it is notable that in all cases, the circuitry is similar. Its form in a generic virtual loop is represented in Fig. 4 and the extended representation in five loops in Fig. 5. The loops are displayed on a posterior–anterior axis in the frontal cortex and also correspond to different and well-identified regions of the striatum, as confirmed by many data reporting the topographical organization of projections and of information representation in these circuits [2, 88]. The loops have been named depending on the frontal areas mainly engaged and consequently on the kind of responses selected by the loops [2]. In the motor loop, the dorsolateral striatum (mainly the putamen) receives information from the motor cortex and proprioceptive information from the sensory cortex. The loop is somatotopically organized, to select different classes of motor actions, e.g. involving the face, the arm or the legs [2]. The oculomotor loop [50] participates in gaze orientation and involves also regions of the dorsolateral sensorimotor striatum (mainly the caudate nucleus) receiving projections from the posterior dorsal cortex together with the Frontal Eye Field FEF frontal area, known to encode gaze movement [107]. These two latter loops, both including the dorsolateral striatum and involved in sensorimotor behaviors, are sometimes called together motor loops.

The lateral prefrontal cortex loop, also called the cognitive or the associative loop, involves the dorsomedial striatum, the hippocampus and associative regions of the posterior and prefrontal cortex and is mainly engaged in cognitive control [57], related to the ability of the prefrontal cortex to manipulate abstract rules, as described below in Sect. 4.3. In the medial prefrontal loop, the ventral striatum (mainly the core of the nucleus accumbens, NAcc) receives sensory information about the needs of the body and the rewarding value of the CS experienced, and is rather involved in the level of ‘wanting’ associated to instrumental learning, with the anterior cingulate cortex in the dmPFC cortex, monitoring performance with regard to the association of responses and reinforcement, to decide for a cost of response (fatigue, risk) adapted to the level of wanting (energizing role of motivation [76]), and the vmPFC possibly adapting the value of the goal to the needs (devaluation). In the orbitofrontal loop, the ventral striatum (mainly the shell of the nucleus accumbens) receives information from the posterior ventral cortex and from the hippocampus, giving sensory details about objects, and from the lateral part of the orbitofrontal cortex, reported to encode the sensory value of objects, typically of the US, and to define the ‘liking’ of objects or their hedonic value (preference) [59]. Altogether, the latter two loops referring to values have been termed the limbic loops, as a reference to the limbic system (also including the amygdala, hypothalamus, hippocampus, also associated to emotions and motivations). This five-loop system is sometimes summarized into three more general loops [88], concerning sensorimotor (motor and occulomotor) control, limbic (emotional and motivational) control and associative (or cognitive) control.

The anatomical organization of the BG has been described by the parallel processing made by these segregated loops but also by a convergence of information [88] due to several characteristics. Within loops, a funneling effect is obvious when the reduction in the size of data flow from the cortical input to the output of the loops in GPi-SNr is considered [53]. Between loops, overlapping schemes can be deduced from several principles, like the spiral principle proposed in [46], where pathways between loops can be observed through dopaminergic projections and through overlapping frontal representations from one loop to the next. This is also the case, considering the participation in the loops of the subcortical structures mentioned in Sect. 3.1 [69]. These structures can be functionally associated, one to one, to the loops, as proposed in Fig. 5, whereas anatomical data suggest a wider scheme, for example with the cerebellum linked to the motor and oculomotor loops [50] or the amygdala linked to orbitofrontal and medial prefrontal loops [17].

As it is proposed in Fig. 5, taking apart the cognitive loop that will be described in Sect. 4.3, each of the other four CBG loops can be seen independently as selecting a response (an elementary action, an integrated behavior or a decision impacting (acting on) internal variables, depending on the nature of information) with regard to its afferent information, and as participating to the answer to one of the four fundamental questions. In the orbitofrontal loop, What is the goal of my behavior? In the medial prefrontal loop, Why should I spend energy satisfying the corresponding need (and up to which level)? In the oculomotor loop, Where is this goal? In the motor loop, How should I behave (which response should I trigger) to get it?

Depending on the complexity of the task and on the richness of the environment, these decisions can be constrained and articulated in different ways. On one extreme, we are in the domain of goal-directed behavior, when there are several answers to each questions, and when loops must interact to find the best global solution: contingencies between local decisions and their consequences must be evaluated and corresponding reinforcements must be compared. This can be associated to the domain of planning [95], with the classical steps of deciding for goal, motivation, strategy and execution, and of backtracking in the hierarchy when one step is impossible. On the other extreme, we are in the domain of habits, where the current state is enough to directly trigger the response with no need to refer a priori to a model of sensorimotor transitions or to the value of the outcome. Many behavioral experiments [86] have shown that both types of learning are present and in competition in the brain and probably that the longlasting learning of the later is dependent on a supervision by the former. It is consequently important to wonder how the rich representations of states and of sensorimotor contingencies needed by goal-directed behavior are built by learning and have been complexified through evolution by developing different kinds of memories, as we evoke now.

## Associated mnemonic processes

### Keeping a memory of past episodes (the role of the hippocampus)

Basically, we have explained above that, to give an ecological meaning to our behavior, our direct sensorimotor capabilities (being able to orient toward an object of interest (the where question) and being able to exploit the object with the body (the how question)) are enslaved by the motivational and emotional analysis of the situation (the why and what questions). At a first level of complexity, this can be performed by subcortical structures (amygdala, PAG and hypothalamus) learning simple pavlovian associations and having strong relations with the ventral striatum.

In the simplest cases, when the goal of the behavior has been identified in the sensory region of the amygdala (BLA) and is directly available for consumption, BLA activates the amygdalar output CeA for pavlovian response and sends also projections to the shell of NAcc for the corresponding consummatory behavior. Anatomical and functional considerations underline how these responses are similar. There is in fact anatomical continuity between CeA and the shell of NAcc with a proposed similar functional organization [19] including strong dopaminergic innervation and projections to the same regions of motor output (including PAG and lateral hypothalamus).

When the target of the behavior is not directly identified, the general class of motivation can give information to energize a preparatory behavior that will result in selecting the target. This is allowed by projections from CeA to the core of NAcc and can result in simple autoshaping or in more complex goal-directed behavior. This view gives to the ventral striatum (or NAcc) a central role at the interface between pavlovian and instrumental learning [65]. That is the reason why it is particularly interesting to remark that, considering more elaborated information that was incorporated to the system along evolution and particularly from birds [111], information of episodic memory originating from the hippocampus is projected to the striatum mainly in its ventral division [120].

In mammals, within the medial temporal lobe generally reported as dedicated to declarative memory, the hippocampus is more precisely associated to episodic memory [114], allowing to remember specific events in their context. Through its input structure, the entorhinal cortex, the hippocampus receives cortical information from the posterior ventral cortex related to the what and why questions (via the perirhinal cortex) and from the posterior dorsal cortex related to the where and how questions (via the postrhinal cortex, also called parahippocampic, depending on species) and aggregate them, including their organization in time [52], in an episode or event [28]. This association of arbitrary information is made possible by the unique recurrent architecture of the hippocampal region CA3 that makes it work as an associative memory, learning very rapidly an event [54]. This recurrent structure appears in birds [111]. In reptiles, the ancestor of hippocampus is just a memory dedicated to spatial information.

Decision to memorize an event can be made intrinsically on the basis of its novelty and from extrinsic afferents, particularly originating directly or indirectly from the amygdala [91], signaling errors of reward prediction and consequently a need for a more precise learning. Errors might be due to ambiguities in the conjunction of features [84] or in their temporal ordering, as the hippocampus is also particularly critical for sequence and delay learning [52]. The dentate gyrus (DG) appears in mammals [111] as an intermediate step between the entorhinal cortex and CA3, with different functions of pattern separation [55] during learning, to avoid errors in recall. In the recall process, thanks to direct projections between the entorhinal cortex and CA3, the hippocampus can be activated from partial information, evoke the complete episode and facilitate reactivation of other brain regions [44] via its output structures, CA1 and the subiculum. This has been for example reported as contextual signals sent to the amygdala for the extinction of pavlovian conditioning [73] or as predictive signals of possible paths sent to the entorhinal and prefrontal cortex and also to the ventral striatum during navigation of rats in a maze [44].

From its ability to store and later detect and recall complex multimodal episodes, particularly including delays between their constituents, the hippocampus provides the ventral striatum and the amygdala with more complex features than simple sensory cues sent by the thalamus or the cortex. It is for example reported that hippocampal inputs are critical to the amygdala in pavlovian trace conditioning [91], when the CS and the US are separated by a delay. This also allows to create conditioned reinforcers in the amygdala, corresponding to subgoals or intermediate steps in a sequence of behaviors, sent to the ventral striatum and evoking surrogates of rewards when the actual reward is distant, as it is often the case in instrumental conditioning [17].

The distinction evoked above between the posterior ventral cortex (the what and why questions) representing perception for recognition and the posterior dorsal cortex (the how and where questions) rather representing perception for action [72], has also been clearly reported in the hippocampus [36], with a dorsal region rather involved in navigation, with neurons coding for location (place cells) or head direction and a ventral region rather involved in emotional aspects and massively projecting to the amygdala and to the ventral striatum (mainly the shell). It must be noted that the dorsal hippocampus also projects to the core of the ventral (and the dorsomedial) striatum and to the anterior cingulate cortex [92], which underlines the special position of the medial prefrontal CBG loop, intermediate between pavlovian and instrumental conditioning and associating basically responses and outcomes. This will be discussed in more details in Sect. 4.3 below.

Recent hypotheses about the interplay between the hippocampus and the entorhinal cortex, associated to the computing formalism of successor representation [109] also propose a powerful basis for reasoning processes. In this view, CA3 is seen as a cognitive map encoding not only locations (classically associated to place cells) but also prediction of possible transitions to other locations under a probability distribution learnt through episodes stored in CA3. It is then proposed that this information is exploited in the entorhinal cortex, where so-called grid cells encoding various metric representations have been reported to perform path integration by performing a hierarchical decomposition of space. Now considering that the hippocampus also learns episodes of non-spatial concepts, the same process will correspond to propose in the corresponding regions of the entorhinal cortex, a hierarchical decomposition as can be observed in planning, extracting subtasks and subgoals at various levels of description [110]. It has been observed for some time [96] that internally generated sequences resembling such episodes are replayed at certain key moments and particularly during rest. This replay mechanism is proposed to reinstantiate retrospective memory in the posterior cortex to improve training. It could also be a basis for prospective memory in the PFC, with such mental processes as planning, reasoning and more generally thoughts, as discussed in Sect. 4.3.

In summary, the hippocampus can represent complex events, corresponding to specific episodes, introducing rich and complex sensory information in pavlovian and instrumental conditioning. This gradient of complexity in sensory inputs, from specific cues encoded in the sensory cortex to cognitive maps and emotional episodes in the dorsal and ventral hippocampus is very nicely illustrated in [120], gathering anatomical information in rats about hippocampal projections to the striatum, the amygdala and the frontal cortex, ordered along that gradient. Such complex information allows birds and mammals to learn pavlovian associations with a complex pattern in time. It is also critical in goal-directed behavior which requires the prospective evocation of perception–response contingencies and of outcome values, as it has been reported in the hippocampus and the ventral striatum [13].

Experiments in rats [86] have shown that rapid and flexible goal-directed behavior involving the hippocampus and the dorsomedial striatum can be replaced by repetition by a habitual behavior involving the dorsolateral striatum and corresponding to a simple stimulus–response association insensitive to reward devaluation. Since the dorsolateral striatum has no hippocampal but only cortical sensory inputs, it can be thought that the slow habitual learning is constrained by the time for consolidation from the hippocampus to the sensory cortex, of the critical events triggering the response, as described in Sect. 4.2. In fact, when habits have been learned, the same experiments [86] show that both goal-directed and habitual learning coexist and are in competition. In a very interesting view [93] using the actor/critic framework where reinforcement learning is decomposed in an actor applying the current policy (rules of behavior) and a critic learning from errors of prediction the value of the outcomes and modifying the policy correspondingly, the dorsolateral striatum is proposed to be the actor for habitual behavior and the dorsomedial striatum to be the actor for goal-directed behavior. The shell, corresponding to the consummatory behavior and learning explicitly the value of the outcome, is proposed to be the critic of the dorsomedial striatum, learning explicitly the model of the world for goal-directed behavior, whereas the core, associated to preparatory behavior not specific of the outcome and learning only to associate a response to a motivational value, should be the critic of the dorsolateral striatum, associating directly in a habitual mode states with responses.

All these pieces of information give a very important role to the ventral striatum at the interface between limbic and motor systems. The ventral striatum is described in [65] as the place where motivational values are assigned to goals from their pavlovian value given by the amygdala and their salience and novelty given by the hippocampus. This results in associations between the outcomes and their motivational value in the shell and between responses and outcomes in the core, and the corresponding energizing effect on instrumental behavior. The dorsomedial striatum is also a key player in instrumental behavior and its role will become more clear as more details are given about the prefrontal cortex in Sect. 4.3.

### Building abstract categories (the role of the posterior cortex)

Beyond the memory of specific episodes in the hippocampus, an important innovation has been brought in mammals by the cortex to build structured high-level information over simple signals: the elaboration of abstract categories composing a semantic memory. In the posterior cortex such a representation is built on data flows corresponding to the sensory dimensions evoked by the four questions discussed above (cf. also Fig. 5). This results in hierarchical cortical areas with neuronal populations responding to more and more complex objects [102], building more and more abstract categories in the ventral information flow relating the exteroceptive and interoceptive poles, for the What and Why questions. Based on considerations on the timing of information propagation [78], the information flow is described as parallel rather than serial in the dorsal pathway to elaborate categories between the exteroceptive and motor poles, related to the questions Where and How, even if intermediate strategies are also observed in associative areas, between a purely constructivist hierarchical and a purely purposive specialized view of information processing [77]. This intricate representation is particularly useful to account for selective attention, a function of the posterior cortex particularly critical in primates [37], associating selection of spatial regions and implicit or explicit (covert or overt) involvement of body parts in the dorsal regions (for example corresponding to eye movements in the visual case) together with an anticipation of the subregion of the sensory space that will be available and the focused processing of critical features in the ventral regions.

In these associative regions, one crucial (and still open) question is about the choice of the compound objects to be represented since the combinatorics is obviously too large for a systematic representation. This selection is made by learning and in an ecological view, a simple (but vague) criterion is: “Those which are the most useful to the organism”. A more precise specification must rely on the mechanisms triggering sensory learning in the posterior cortex [1], including the role of cholinergic modulation triggered by the amygdala, in case of error of prediction, to favor attentional process in the cortex [90], and the role of reinstatement (or replay) of episodes in the cortex, driven by the hippocampus in the consolidation process [67].

Another important actor in the processes described in this section is the sensory thalamus [106], for the critical role of its sensory part in the activation of the posterior cortex, conciliating feed-forward sensory input and feed-back cortical expectations, and also in cortical learning of new categories, particularly involving multimodal features. Nevertheless, it will not be described in details in this paper. Nor will be described the motor thalamus, even if it also has a critical role in the functioning of the frontal cortex presented below.

### Building flexible sequences (the role of the frontal cortex)

The organization of the frontal lobe can be described in reference to regions of the posterior cortex in which frontal regions can control transitions of states [16]. In the motor cortex, neurons arranged in stripes symmetrical to the somatosensory cortical area can trigger elementary motor actions modifying the position of the bodily scheme until the sensory goal of the action (e.g. position of a limb, characteristics of the sound produced by the phonatory apparatus) is reached. Motor control is also reported in the premotor cortex, with a more integrated topography [42], corresponding to more ecological categories of the behavioral repertoire like climbing, reaching, etc. Similarly, in oculomotor regions like the frontal eye field FEF [107], transitions are between initial and final targeted eye positions.

The same process of control of transition between present and targeted states can be used to describe the functions of the limbic frontal regions. In the orbitofrontal cortex, lateral and caudal regions have been described [87] as learning sensory features of rewarding stimuli and ordering them in a transitive way to define preferences for emotional stimuli, seen as potential goals of the behavior. This is the basis for emotional control, where the selection of a desired goal is sustained until that goal is obtained. Complementary to this consummatory behavior built on specific sensory features, the preparatory behavior can be organized in the medial prefrontal cortex on more general properties, with the ventromedial prefrontal cortex evaluating the rewarding value of stimuli for decision making and, in the dorsal part, the anterior cingulate cortex performing motivational control [58]. Basically, this region, described as associating responses to outcomes, is in charge of deciding if the energy required by the responses selected in the preparatory phase is worth the corresponding need. Accordingly, it is reported to energize the behavior, i.e. to evaluate up to which level it can be engaged and, when a strategy is selected, to maintain this selection until it is achieved (or given up). Here, also motivational control can be defined as a transition from selection to satisfaction of the need, with maintenance of activity if not achieved.

It can be remarked that the medial prefrontal cortex is structured in a ventral part, deciding for the selection of a goal from its rewarding value and current motivations, and a dorsal part, selecting the response from its cost. The dorsal part also monitors the progress of the actual behavioral sequence [51] and, by comparing actual and predicted costs and rewards, is able to detect errors and conflicts indicating that the current control is not adapted. This can lead to direct adaptation of the motivational control or when this adaptation is not trivial and requires elaborated contextual rules, the needed cognitive control recruits additional circuits in the lateral prefrontal cortex [5]. This region, increasingly large in primates, is also distributed in ventral and dorsal regions and is reported to elaborate complex rules (useful to address a specific task), with their complexity defined as the level of sequential arrangement of responses in the dorsolateral prefrontal cortex and as the level of precision in the definition of cues in the ventrolateral prefrontal cortex [81, 83], both together able to build a complex strategy, decomposing a goal and a level of engagement into subgoals and responses to get them. The same principle of maintenance of activity until satisfaction (or giving up), described as a working memory process [39], results in resistance to distraction, another strong characteristic of the prefrontal cortex. Altogether, it has been proposed that the anterior cingulate cortex plays here a central role [105] by integrating from the orbitofrontal cortex, the ventromedial prefrontal cortex and the lateral prefrontal cortex three different factors (respectively, the value of the reward, the cost of effort and the cost of cognitive control), to determine whether, where and how much control to allocate.

Three different levels of reasoning have been proposed in mammals, along evolution [56], relying on different kinds of associations learned in different cortical regions. In a first stage, rodents are able to learn to select the most rewarding response from the perceived stimuli and to correct errors. In their motor loops, they learn S–R associations, called the selective model, useful in the habitual behavior and for anticipating sensory states resulting from a response. Rodents are also able, thanks to their limbic loops, to predict the forthcoming outcomes and to adapt their future behavior in case of errors. These S–O associations are called the predictive model (and are similar to what is learned in pavlovian conditioning). In a second stage, thanks to their lateral PFC as evoked just above, primates can build more complex criteria to select a behavior adapted to specific contexts and to adapt their behavior to the specific situation (context) before committing errors. They accordingly build a contextual model and define rules adapted to specific contexts. In the third stage, according to [56], the frontopolar cortex allows humans to monitor several strategies in parallel and to perform hypothesis testing independently from the actual behavior, thanks to prospective mechanisms as described in Sect. 4.1.

Some generic mechanisms of the frontal cortex can be re-interpreted now. Each region of the frontal cortex has been described as preferentially linked to a posterior cortical region and composed of responses monitoring a transition from one state represented in this posterior region to another (from an initial to a final position; from need (e.g. water deprivation) to satisfaction of the need (satiety), etc.). This can be interpreted with the scheme S1–R–S2, where S1 is the initial condition eliciting R as a possible response (cf. [79] and the principle of affordance) and S2 is the consequence that can be anticipated if R is preactivated. Conversely, if S2 is a desired state, R is the response that has to be activated to obtain S2, which is possible if S1 is compatible with the current state. Else, R can display a sustained activity, as in working memory, and remain actively waiting until S1 is satisfied. This interpretation is reminiscent of behavioral studies where antecedents and consequences of goal-directed behaviors are seen as beliefs and desires [8] and of more theoretical works on planning [16, 95] explaining how goals (desires) can be decomposed into subgoals (S1 becomes desired) and recursively executed in such S1–R–S2 schemes. In our view, these intermediate steps with subgoals can also be provided by the hippocampus and the entorhinal cortex at various levels of description, as suggested in Sect. 4.1 for prospective memory. They are executed by cognitive control in lPFC: the goal remains active as a working memory in mPFC and activates subgoals and means (which can be seen as intentions) to get them in lPFC until good conditions are met (e.g. finding the kitchen seen as a subgoal to open the fridge), without forgetting the initial goal (of drinking a bottle of water), as ensured by the sustained activity insensitive to distraction.

This view is very consistent with an interpretation of the role of the BG for the dynamic gating of frontal representations [81, 83], switching from the updating of the choice of the best response to be selected (from the prediction of the value of its consequence) to the maintenance of its sustained activity until this consequence is obtained. This also explains why goal-directed behavior is defined by its sensitivity to goal devaluation and contingencies of responses [8]: in the habitual mode, S1 directly triggers R with no consideration of S2 and of the value of the goal obtained at the end of the process whereas in a goal-directed process, when a action is executed, its consequences are compared with its expected results and the corresponding contingencies are updated in case of a mismatch. Beyond real actions, the premotor theory of attention [101] proposes that attentional control is a weaker activation of motor control, allowing to explore the same situations by an access to the same learnt representations with no (or covert) action. The situations evoked above can consequently be examined in such a mode, corresponding to virtual thoughts instead of real actions in the world.

Globally, this heavy and structured process of the frontal (= prefrontal + premotor and motor) cortex can be summarized as follows: Exteroceptive and interoceptive stimuli can elicit response preactivations in the motor and limbic prefrontal cortex which can also evoke the anticipated consequences in exteroceptive and interoceptive terms. In simple and stable worlds, the elaborated model of the world can become of good predictive quality and at the end, the initial stimuli can be sufficient to trigger directly responses without evoking their consequences. This corresponds to the habitual mode, progressively shifting the control from the limbic to the motor loops [49] and in the long term, only mobilizing the motor cortex in a basic stimulus–response scheme.

Nevertheless, in the early phases of learning or when the world is changing or when the best behavior to be selected does not correspond to the most frequent (for example in a specific context), a more precise analysis of the recent history of performance must be carried out, involving the limbic parts of the prefrontal cortex and of the basal ganglia. This is the reason why the dorsomedial prefrontal cortex is often reported to be involved in error detection and conflict monitoring [103] and the ventromedial prefrontal cortex to be sensitive to devaluation of outcome [59] for example in case of reversal and extinction. The interoceptive preactivation of the limbic loops can evaluate and supervise this goal-directed learning, depending if gains or losses are observed between anticipated and actually obtained punishments and rewards, and results in the selection of the current goal and motivation.

In this goal-directed process, the role of the basal ganglia is prominent as a critic in the limbic loops to learn from errors of prediction and as an actor to explicitly trigger step by step the full plan of responses, as it was explained above. Concerning the transition between loops, note that both ventro- and dorsomedial prefrontal cortex project to the dorsomedial striatum [44] and that the exteroceptive preactivation of the motor loop is critical to offer affordances that help select the most appropriate preparatory behavior [95], also supposed to be performed in the striatal region. The double role of the dopamine [15] must be also particularly emphasized here. On one hand, dopaminergic signals carry reward prediction errors that can be used for learning as it has been shown for a long time in reinforcement learning, with dopaminergic projections from VTA to the ventral striatum mainly for pavlovian aspects and from SNc to the dorsomedial striatum for the instrumental aspects [122]. These pathways are also at the basis of the spiral principle by S. Haber evoked above [46], concerned with the articulation between CBG loops. On the other hand, dopaminergic projections from VTA to the PFC participate to the modulation of performance, by acting on the gating mechanism between maintenance and updating of activity in PFC [80] in case of sudden changes in goal representations. This dual role of dopamine can also be seen as a dual contribution to, respectively, model-free and model-based reinforcement learning.

In a classical view (cf. for example [25]), goal-directed behavior is associated to model-based reinforcement learning and habitual behavior to model-free reinforcement learning, in reference to computational learning mechanisms where contingencies of the world useful for decision are, respectively, gathered in an explicit model of the world or cached in variables summarizing the current state. The analogy refers to the fact that cached variables propose a more compact and less-expensive representation than an explicit model and are less sensitive to accumulated approximations, and that in contrast, they are very long to evaluate and to modify when the world changes. But the analogy has also been recently questioned [71]. Model-free is not a perfect term, since a model has been built and even if it has been compiled in cached variables, they ultimately depend on rewarding values, accordingly, value-free (total independence on reinforcement should be the good term for habits. Concerning model-based learning and the manipulation of explicit knowledge, such information can come from the temporal cortex (for semantic memory or from the hippocampus (for episodic memory. Experiments have shown that the ventromedial prefrontal cortex is a key relay to associate this information in the process of cognitive control [117].

We have evoked above selective, predictive and contextual models learned in the motor, limbic and lateral loops of the frontal cortex by accumulating history of occurrences of corresponding motor, sensory and rewarding cues. Altogether, this has led to propose the concept of Task Set to describe the organization of frontal regions [104] and to propose computational mechanisms for the coordination (cognitive control) and selection (decision making) of thoughts and responses for adaptive behavior [30]. In the ideal case, a configuration of cognitive processes in these loops has been selected as the behavior adapted to the situation and it will be actively maintained for subsequent task performance. In case of a problem, some processes will be adapted a posteriori and a new more adapted configuration will be selected or possibly created. These mechanisms are reported to be compatible with the observed arrangements and activations of frontal regions [31], they still have to be consolidated at the computational level beyond stereotyped tasks, particularly concerning the question of creativity [30]. In the current view, applying the best Task Set from a previously learned repertoire rather suggests a strategy globally similar to model-free learning, with critical points where the strategy must be explicitly reconsidered in a model-based manner. In this insightful view, it has to be noted that model-based and model-free approaches are cooperative and not concurrent, thus minimizing their reported weaknesses. Such integrated architectures have already been proposed in the past in reinforcement learning (see the Dyna architecture in [108]) and are presently extended with stronger biological bases [47].

All put together, we are still under the double constraint of goal-driven and stimulus-driven behaviors, with the general pre-eminence of the limbic side [82] generating needs corresponding to motivations to be fulfilled. These motivations are then translated into desired goals to obtain from the environment. Reciprocally, stimuli can preactivate motor responses by affordance, which will be directly triggered in case of habits and which will otherwise simply generate predictions of what could result of the response if triggered. In a pavlovian scheme, stimuli can also preactivate anticipated rewards. From this common basis, agranular frontal areas (motor, premotor and lateral orbitofrontal cortex) can directly make a pertinent decision if the world is stable enough. Else, cognitive control is needed with the help of medial and lateral prefrontal cortex, inhibiting the default behavior and imposing new rules adapted to the context, by an attentional process on the posterior cortex. Within this view, the behavior is seen as the control of perception, with affordances to select responses, depending on the desirability of the predicted outcomes [97]. Interestingly, concepts like intentionality, thoughts and imagination (seen as a control of thoughts can also be evoked in that view, as mechanisms of the cognitive control, higher cognition is in fact partly elaborated on the same basic sensorimotor and motivational loops.

## Discussion

In this paper, we have proposed a systemic description of the brain, as a contribution to a brain theory and as a general framework where specific models in computational neuroscience should be positioned before their development. Beyond its intrinsic interest, this framework is necessary, else the risk is to build models of particular neuronal structures in isolation without reference to more global information flows and cognitive functions and consequently to neglect some characteristics of the structure or to overload it with functions carried out in other parts of the cerebral network. This description in width rather than in depth also evokes a variety of sensorimotor loops and levels of representation, from pavlovian to instrumental conditioning, from goal-directed to habitual behavior, from episodic to semantic memory, from simple to complex rules, that can coexist and act in competition or in synergy. Having a global view of the underlying information flows can be useful to set a specific model back in a more general and dynamic cognitive context.

**Fig. 6:**
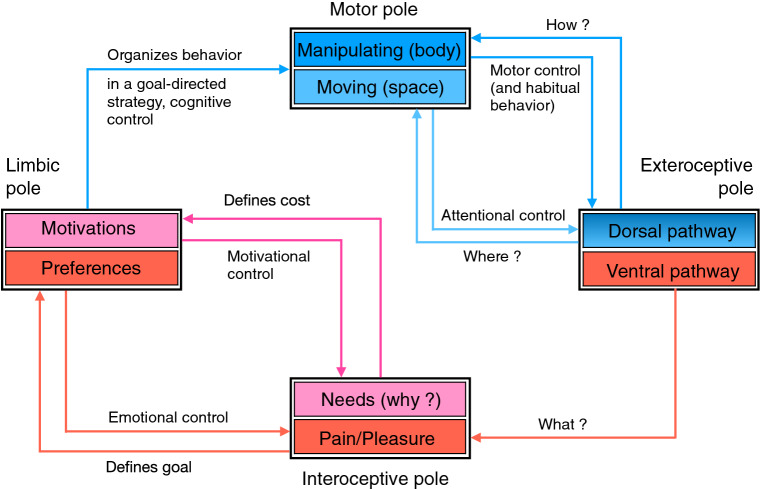
Integration of the presented enactive and functional views. This figure integrates the enactive view of the brain–body–environment system and the functional view of brain structure in a behavioral organization, where sensory interoceptive and exteroceptive poles interact with the limbic and motor poles to decide for the main characteristics of the behavior. Basically, addressing the four fundamental questions results in specifying sensory constraints in the motor pole related to the position in space and to the body, and in the interoceptive pole related to pain and pleasure and to fundamental needs. This will define preferences and motivations in the limbic pole, generating directly a consummatory behavior or organizing a preparatory behavior with the motor pole that can particularly trigger movements and evoke selective attention to obtain desired changes in the internal and external world and, accordingly, in the sensory perceptions. Consequently, the general question of control raised in Fig. 1 must be divided up into motivational control monitoring needs, emotional control deciding for goals, attentional control selecting them in the environment, cognitive control organizing properly the behavior in goal-directed strategies and motor control triggering each step in that strategies and for immediate response in habitual behavior. Colors in the picture refer to the colors used in Fig. 5 to evoke fundamental questions

This framework considers several fundamental aspects of the brain, seen as the device controlling the behavior of the body, as summarized in Fig. 6: (i) In an enactive view, the brain has to elaborate loops with the internal and the external environments (cf. Fig. 2) and to ensure their stability for the general goal of survival. This circular causality has already been expressed in many systemic views including, in computational neuroscience, a very interesting approach exploiting the powerful formalism of thermodynamics [38]. It confirms also the fundamental organization of the brain in sensorimotor loops and structures [45] which has already been mentioned as central to organize behavior and even to define consciousness [27, 34, 64]. (ii) To provide a more precise account of the various characteristics of behavior, we have structured brain functions as answering four fundamental questions (what, why, where and how). From the basic what/where system [115], this terminology has a long history in neuroscience, including more recent and precise views [72, 81, 83]. In particular, a very close formulation is proposed in [119] but is less accurate concerning the mapping to brain structures and functions. This latter paper insists on the need for the organism to answer these four fundamental questions (and also the When question, which is implicitly tackled in the present paper) but they are not related as it is the case here to specific regions of the striatum, frontal and sensory cortex and to specific subcortical structures. (iii) Functional and anatomical considerations are, respectively, added in Figs. 4 and 5 to explain how these questions can be addressed in brain structures, particularly taking phylogeny into account. Basal animals have the same fundamental problems of behavior organisation for survival; the main difference is about the quantity and quality of information that is provided and built in internal representations to define goals and needs and to elaborate answers. In this perspective, the mnemonic mechanisms described in Sect. 4 are seen as new representations provided by phylogeny to extend the power of the same pavlovian and instrumental mechanisms by applying them on past episodes and prospective cases (episodic memory), new categories (semantic memory) and flexible rules (working memory). The transformation of memory systems along evolution is presented in details in [74], in particular with more information about their impact from a social point of view in humans but with no reference to computational aspects which are central here.

Elaborating such a framework also provides opportunities to put together many facts at various levels of description and to elaborate on them principles of brain functioning and of cognitive architecture. Basically, the behavior is elaborated from cross-talk between the motor and limbic poles (cf. Fig. 6). The motor pole receives affordances from the exteroceptive pole that might result in directly triggering habits or at least preactivating some responses. The limbic pole receives emotional and motivational information from the interoceptive pole which can directly trigger pavlovian reflexes and consummatory behavior or lead to the definition of goals and motivations. Then, instrumental conditioning will allow to specify a more appropriate preparatory behavior, based on preactivated responses and learned contingencies. In the simplest case, as permitted by the agranular frontal areas, an immediate decision is sufficient to trigger the behavior in a stable world, from the selective and predictive models. Else, as permitted by granular frontal areas, the sensory representation must be modified by cognitive control to be adapted to the situation, after an internal deliberation possibly exploiting episodic and semantic memory. What makes the picture difficult to comprehend here is that all these memory systems coexist, collaborate and compete for the control of behavior. This has been for example studied for pavlovian and instrumental conditioning [32] and for model-based and model-free learning [24], each time mentioning the dorsomedial prefrontal cortex as the place where the associations are weighted, depending on the characteristics of the predictive models and of the present situation.

As we might expect, this sketch gives a prominent role to pavlovian and instrumental conditioning, in this survival-oriented definition of behavior. Nevertheless, the contribution of other kinds of learning is obvious, specifically related to semantic memory in the posterior cortex, episodic memory in the hippocampus and working memory in the frontal cortex. More fundamentally, this also lays emphasis on the fact that cognition can be described as a dynamical system of interacting memories, some acting to provide information to others, to replace others when they are not efficient enough, or to help for the improvement of others. Such principles have already been described with a very strong impact in improving our understanding of cognitive mechanisms [67]. The framework that we have proposed here is a very convenient tool to study such mnemonic synergies, very difficult to delimit because not related to a unique cognitive function. We have for example seen that goal-directed behavior can generate a prospective memory (also called “memory of the future” [39]), as it is observed for places in the hippocampus and for rewards in the ventral striatum, and can also generate a retrospective memory that can participate in training the habitual system [13]. Precisely understanding how these processes work and interact is an important challenge for future research.

It can also be remarked that, throughout the described mechanisms and representations built in the various kinds of memory, a central role is given to sensations. Apart from motor actions participating to the selective model (learning the S1–R–S2 associations that might drift toward the S-R habitual schemes), all the other learned structures are essentially related to sensations. This corresponds to the S–O predictive model but also, within the contextual model, to the idea that internal responses are going to bias sensory representations by attentional means. This is consistent with the idea of motivational priming [11] proposing that motivations are in fact oriented toward sensory features, and more generally with the principle that behavior is the control of perception [95].

Several other brain theories have been mentioned in this paper, including mechanisms and principles consistent with the present framework. This is particularly the case for interacting memories and the Complementary Learning System [67], though limited to episodic and semantic memories, and for the central role of CBG loops and major contributions of R. O’Reilly’s group, notably [81, 83] and [82], though an integration of the proposed mechanisms in a global framework is still missing. A global brain model, associated to a general computing framework called NEF (Neural Engineering Framework) has been proposed in [35], including very similar principles and the central role of CBG loops, but learning principles are more elementary and less related to the view of incremental learning. Another consistent global brain model has been proposed, centered on the Global Workspace hypothesis [27, 34], giving to the dorsolateral PFC a central role in consciousness, in association to the other CBG loops, but provides less details about other phases of behavioral control and about the role of subcortical structures. In spite of these differences, it is important to mention that all these brain theories are compatible with the present framework, which clearly borrows from them and proposes an integrated view of their principles.

Describing the brain as an architecture of learning systems has also strong implications in Machine Learning, which has been focused in the recent years on the paradigm of Deep Networks, powerful to implement single tasks, but lacking flexibility in several aspects. Concerning extensions to the processing of structured and temporal data, architectures like the Neural Turing Machine and the Long Short Term Memory have been proposed [41] but they still rely on the same learning principle using differentiable functions, known to be very slow and data consuming. Considering bio-inspired principles to adapt Deep Learning architectures to more realistic time and volume of learning is consequently needed [33]. Similarly, it has been observed that classical Deep Reinforcement Learning is slow and not compatible with observations of biological systems in the same tasks, leading to the definition of episodic reinforcement learning and meta-reinforcement learning [14], specifically built with inspiration from, respectively, the hippocampus and the prefrontal cortex [121]. What is still lacking and could benefit from the present framework is the way both learning methods are associated and interact in their development.

In addition to the definition of interacting mnemonic synergies as a basis to ensure really autonomous learning, which is very poorly addressed in classical Machine Learning, a more realistic view of pavlovian and instrumental conditioning can be very precious to revisit classical Reinforcement Learning. Such a contribution is for example proposed in [6] where the selection of responses is controlled by a utility function, defined by a weighted combination of value and risk. This can extend the classical actor/critic architecture and needs to be deepened, since many questions remain about the exact location of the critic (if any) and about the validity of the hypothesis of a double model-based and model-free actor-critic system evoked above [93]. This hypothesis, compatible with the proposed framework, could be investigated more deeply in neuroscience, based on precise predictions about the roles of the corresponding CBG loops as discussed in [75].

Our systemic framework is also useful, because it helps revisit the role of certain cerebral structures. Particularly, it appears from our analysis that the BG is best defined as a modulatory system that provides adaptive gating signals to the frontal cortex, instead of the prevalent ideas that BG directly encodes S-R associations and can be defined as a procedural learning system. This is confirmed by the (relatively) low impact of BG lesions in behavioral performances but rather in learning [86]. One step further, the following phylogenetic interpretation could be proposed: beyond simple reactions due to ancient subcortical structures, the hippocampus and the BG could be proposed as two structures endowed with rapid learning to adapt the behavior to specific cases, respectively, corresponding to emotional episodes and to behaviors where the motivation must be explicitely reminded. But in both cases, the goal of the organism is to identify and learn criteria to automatize the behavior. In the same way as there is a consolidation from the hippocampus to the posterior cortex to create new categories in semantic memory, appropriate to discriminate the world, the transfer from goal-directed behavior involving the BG to habits in the motor pole of the frontal cortex might be seen as a way to create motor routines, giving a behavioral repertoire adapted to our needs. At the end, this would result in a system parallel to the ancient subcortical structures, except that the sensory and motor characteristics would have been selected and learned in a slow process, from interactions with the world.

Our framework also indicates some specific structures, which can be seen as a kind of hub in the cognitive architecture, because they are fundamentally multimodal and coordinate transfers between memory systems. For different reasons, this is particularly the case with the ventral striatum and the hippocampus which would have to be studied more deeply in that perspective. Another domain which remains not detailed enough is the precise definition of the mechanisms of cognitive control, with the elaboration of complex rules in the lateral prefrontal cortex and their specific dorsal and ventral aspects. Preliminary theories have already been proposed [5, 57] and should be more deeply explored in specific behavioral applications for a better understanding. Similarly, the role of neural structures like the frontopolar cortex or the thalamus and of neural mechanisms like neuromodulation should be more precisely studied and associated to the proposed framework. Testable predictions related to the latter mechanism have been already proposed in [3].

It might finally be said that this paper proposes a very “mechanical” view of the brain, neglecting highest cognitive functions of the brain, for example related to social aspects, language, not to evoke internal thought, mind wandering and consciousness. Based on experimental works in connectomics studying brain networks, we have recently proposed that cognitive functions like creativity could be studied with such a brain model, providing also testable predictions [4]. More generally, we think in fact that most of these functions rely on the same bases as the ones evoked here, since they also correspond to organize behavior in time and associate constraints emerging from internal and external worlds because, at the end, the motivation of a human being is also being well adapted to its mental, linguistic and social world.

## Data Availability

Not applicable.
